# Should Burnout Be Conceptualized as a Mental Disorder?

**DOI:** 10.3390/bs12030082

**Published:** 2022-03-17

**Authors:** Lindsey Nadon, Leon T. De Beer, Alexandre J. S. Morin

**Affiliations:** 1 Substantive Methodological Synergy Research Laboratory, Department of Psychology, Concordia University, Montreal, QC H4B 1R6, Canada; lindsey.nadon@mail.concordia.ca (L.N.); alexandre.morin@concordia.ca (A.J.S.M.); 2 WorkWell Research Unit, Faculty of Economic and Management Sciences, North-West University, Potchefstroom 2531, South Africa

**Keywords:** burnout, mental disorder, syndrome, diagnostic, conceptualization

## Abstract

Burnout is generally acknowledged by researchers, clinicians, and the public as a pervasive occupational difficulty. Despite this widespread recognition, longstanding debates remain within the scientific community regarding its definition and the appropriateness of classifying burnout as its own pathological entity. The current review seeks to address whether burnout should (or could) be characterized as a distinctive mental disorder to shed light on this debate. After briefly reviewing the history, theoretical underpinnings, and measurement of burnout, we more systematically consider the current evidence for and against its classification as a mental disorder within existing diagnostic systems. Stemming from a lack of conceptual clarity, the current state of burnout research remains, unfortunately, largely circular and riddled with measurement issues. As a result, information regarding the unique biopsychosocial etiology, diagnostic features, differential diagnostic criteria, and prevalence rates of burnout are still lacking. Therefore, we conclude that it would be inappropriate, if not premature, to introduce burnout as a distinct mental disorder within any existing diagnostic classification system. We argue, however, that it would be equally premature to discard burnout as a psychologically relevant phenomenon and that current evidence does support its relevance as an important occupational syndrome. We finally offer several avenues for future research, calling for cross-national collaboration to clarify conceptual and measurement issues while avoiding the reification of outdated definitions. In doing so, we hope that it one day becomes possible to more systematically re-assess the relevance of burnout as a distinctive diagnostic category.

## 1. Introduction

Described as a modern affliction [1] of epidemic proportions [2], burnout has evolved from being uniquely associated with human services employees to being considered as a relevant phenomenon to consider across all occupational groups [3]. First defined in the 1970s as a state of exhaustion resulting from excessive job demands [4,5], burnout has since gained global traction as a psychosocial adaptation problem recognized by researchers, professionals, and the public at large [6]. Along with this joint recognition, widespread public discourse, and extensive publications, a debate remains regarding what burnout really is and whether it should be considered a distinct psychopathological entity [7,8,9]. For instance, some position burnout as a clearly defined mental disorder and report prevalence estimates reaching as high as 67% among medical populations [10]. Others, however, actively dispute the notion that burnout forms a distinctive mental disorder, insisting that burnout is not a diagnosable condition and, therefore, that any prevalence estimates are nonsensical [11,12]. Indeed, without clear diagnostic criteria, prevalence estimates remain doubtful. In line with this second perspective, psychology researchers have criticized burnout for its shaky theoretical roots, measurement problems, and lack of clinical utility [13]. Despite these criticisms, burnout research has remained widespread, and burnout itself is now, in some countries, close to becoming a legitimate medical diagnosis [14]. Ultimately, we are left with a stark contrast between the cultural significance and widespread recognition of burnout relative to its disputed nature among scientific communities [8]. To shed light on this issue, this study considers whether burnout should, or could conceivably, be characterized as a mental disorder.

## 2. Burnout: A Brief Introduction

Burnout is broadly understood as a psychological state of exhaustion stemming from persistent exposure to work-related stressors while lacking sufficient resources to efficiently cope with these stressors [15]. More precisely, burnout is typically operationalized as a work-related syndrome encompassing at least three distinctive, yet interrelated, components: Exhaustion, cynicism (or depersonalization), and a reduced sense of professional efficacy [16]. When individuals experience burnout, work that was once seen as challenging and meaningful becomes increasingly unfulfilling and unsustainable over time [16].

### 2.1. History

Freudenberger first introduced the term ‘burnout’ to the scientific community based on his observations of employees working with chronic drug abusers [4] and other clinical populations [5]. However, Freudenberger’s work remained autobiographical and qualitative in nature, seeking to recognize, treat, and prevent burnout, rather than empirically evaluate it [6,17]. Interestingly, based on a series of interviews conducted among human service workers, Maslach [18,19] simultaneously noted that many of these employees experienced high levels of emotional exhaustion, displayed cynicism in relation to their work and patients, and felt that their professional competence was compromised. Maslach [19] also coined this set of psychosocial adaptation difficulties “burnout” and extended her initial observations by using qualitative data derived from interviews to develop a self-report questionnaire called the Maslach Burnout Inventory [20].

The 1980s brought widespread recognition of burnout as a preoccupying societal problem, with severe consequences for organizations and employees alike [16]. While clinical psychologists attempted to classify burnout as a mental health problem resulting from maladapted individual stress responses, social psychologists rather emphasized the interpersonal interaction between workers, service recipients, and coworkers as possible drivers of burnout [16]. The development of the MBI marked a turning point in the conceptualization and measurement of burnout by providing a common vocabulary and catalyzing a cascade of quantitative research seeking to increase our understanding of the nature and implications of this relatively “new” psychological construct [16,21].

In the 1990s, burnout research began to extend beyond the human services sphere and researchers started to investigate the individual and organizational drivers of burnout among distinct occupational groups [16] This led to the development of a more generic version of the MBI, the MBI-General Survey (MBI-GS), a context-neutral instrument suitable for assessing burnout across all occupational groups [22,23]. From this point forward, burnout research exploded. A quick search conducted on 26 January 2022 using the PsycInfo database with the keyword “burnout” identified a total of 16,715 publications, following an upward trajectory: (a) 8 publications in the 1970s; (b) 1046 in the 1980s; (c) 1607 in the 1990s; (d) 3463 in the 2000s; (e) 8211 in the 2010s; and (f) 2380 since 2020.

### 2.2. Theory

Maslach et al.’s [16,20] conceptualized burnout as a psychological state encompassing exhaustion, depersonalization (or cynicism), and a reduced sense of professional efficacy. These manifestations are also expected to occur in sequence. First, exposure to work-related stressors progressively lead employees to become exhausted as their resources are overextended to cope with job demands. Second, this exhaustion leads them to emotionally disengage from their work and from the recipients of their care (i.e., depersonalization/cynicism) to protect themselves from further resource depletion [16,24]. Finally, exhausted and emotionally withdrawn employees progressively develop a reduced sense of professional efficacy as they come to realize that their coping efforts interfere with their performance [3,16,25].

The Job Demands-Resources (JD-R) model [15,24,26,27] provides a broader theoretical framework that has helped to generalize burnout research outside of the human services area, under the assumption that the two core components of burnout (exhaustion and cynicism) occur in other occupations. This model posits that (1) exposure to excessive job demands leads employees to develop chronic feelings of exhaustion, while (2) the lack of necessary job resources (e.g., autonomy, feedback, social support, growth opportunities) leads them to progressively withdraw and disengage from work (i.e., cynicism). This JD-R health impairment process further assumes that burnout should in turn lead to a range of physical and mental health problems. Thus far, meta-analytic studies have largely supported the assumptions of the JD-R model [28,29,30].

### 2.3. Predictors

After decades of intensive research, burnout is acknowledged as a critical occupational problem [8] with reasonably well-documented predictors. Thus far, research suggests that most work-related risk factors for burnout can be grouped under six major categories [16,31]: (a) excessive workload or job demands; (b) lack of control or autonomy at work; (c) extrinsic and intrinsic rewards inconsistent with expectations, or a lack of rewards or recognition at work; (d) a lack of social integration at work (i.e., support, closeness, teamwork); (e) a lack of fair and respectful treatment at work; (f) a lack of fit between one’s personal values and those of the workplace.

Beyond these work-related characteristics, some individual risk factors also seem to play a role in the emergence of burnout [32]. In a meta-analysis of 114 studies, Alarcon et al. [33] noted that some personality characteristics (i.e., high neuroticism, high introversion, low conscientiousness, and low agreeableness) were consistently associated with burnout. In particular, neuroticism seemed to be more strongly associated with exhaustion and depersonalization (or cynicism), whereas introversion seemed to be particularly relevant to the emergence of a reduced sense of professional efficacy. These associations seemed to be maintained longitudinally [34]. Alarcon et al. [33] also reported significant associations between burnout and lower levels of self-esteem, self-efficacy, dispositional optimism, and hardiness (i.e., the ability to tolerate stressors), as well as with a more external locus of control. By highlighting the benefits of feeling in control, this last set of results reinforces the role of work characteristics interfering with employees’ autonomy at work as critical drivers of burnout. Importantly, tentative evidence suggests that personality may play an even stronger role than work-related characteristics in burnout development [35].

### 2.4. Consequences

Burnout has been found to be associated with a wide range of maladaptive outcomes for employees, service recipients, and organizations alike [3]. Notably, employees reporting high levels of burnout are more likely to experience physical and psychological health problems, including sleep disturbances, headaches, and infections [36], as well as higher levels of depression, suicidal ideation, anxiety, and life dissatisfaction [37,38]. As a result of these health-related implications, burnout also tends to be associated with more prolonged sickness-related absences and higher turnover rates [39,40]. Furthermore, burnout has also been found to interfere with employees’ work performance, in turn negatively impacting service recipients [41], patient care [42], clinical reasoning [43], and even student achievement [44,45]. At the organizational level, these various consequences add up, forcing organizations to invest additional resources to support their employees while maintaining the quality of their services [46,47].

## 3. Burnout Measurement

To this day, the MBI-GS remains the most commonly used instrument for measuring burnout [6,9,48]. The MBI-GS is a context-neutral burnout questionnaire, comprising 16 self-report items, rated using a 7-point response scale, and measures the 3 dimensions of burnout (exhaustion, cynicism, and reduced professional efficacy) as conceptualized by Maslach et al. [16,20]. In line with theory, the exhaustion scale measures the experience of being overextended and depleted, the cynicism scale reflects distancing from one’s work, and the reduced professional efficacy scale focuses on one’s perceptions of their ability to be effective and productive at work (reversed scale).

Over the years, psychometric research has revealed that the reliability of scores obtained on the MBI tend to vary significantly across studies as a function of the specific sample, linguistic version, and MBI version being considered. For instance, Wheeler et al. [49] meta-analyzed 98 studies to examine the scale score reliability of the MBI and found that, whereas responses to the emotional exhaustion subscale had a Cronbach’s alpha averaging 0.87, responses to the cynicism/depersonalization and reduced professional efficacy subscales had significantly lower, albeit acceptable, alphas averaging 0.71 and 0.76, respectively. In terms of factor validity, many studies have supported that responses to the MBI are best represented by three factors (each of the a priori dimensions), equivalent (i.e., measurement invariance) as a function of sex/gender, type of sample, and linguistic version [48,50,51]. However, alternative factor structures (e.g., one-factor, two-factor, and bifactor) have also been supported [52,53]. Furthermore, a recent systematic review suggests that the psychometric properties of scores on the MBI remain low relative to other less common burnout measures [54]. Moreover, the MBI has also been frequently criticized for the wording of its items, whereby all exhaustion and depersonalization (or cynicism) items are worded negatively, while all professional efficacy items are worded positively. This design can result in artificial factor solutions, with positively and negatively items naturally clustering together [15,55]. In fact, Bresó et al. [56] found that a negatively worded professional efficacy scale (i.e., inefficacy) performed better than the original. In contrast, others have reported that the original, positively worded professional efficacy scale might tap into a different psychological construct relative to the other subscales [53,57,58].

This criticism, in part, sparked the development of the Oldenburg Burnout Inventory (OLBI) and other less common measures of burnout. Given our focus on whether burnout should be considered as a mental disorder, we limit our presentation to the most common measures relying on the currently dominant operationalization of burnout (for a psychometric review of other measures, see [54]). Among the measures excluded from this presentation, the Shirom and Melamed [59] burnout measure is noteworthy, as it relies on a slightly distinct operationalization of burnout focusing on the dimensions of cognitive weariness, physical fatigue, and emotional exhaustion). Coming back to the OLBI, this instrument was developed alongside the job demands-resources (JD-R) model as an alternative way to measure burnout beyond the human services domain while addressing some of the limitations of the MBI [15,26]. The OLBI includes 16 positively and negatively worded items designed to measure two respective continuums ranging from: (1) exhaustion to vigor (i.e., the exhaustion dimension) and (2) disengagement to dedication (i.e., the disengagement dimension, corresponding to the depersonalization/cynicism facet of the MBI). The OLBI further expands on the MBI by incorporating cognitive and physical components of exhaustion, rather than solely measuring emotional forms of exhaustion [15,26,60]. The OLBI has been praised for its strong theoretical roots and psychometric properties, for addressing wording-related limitations inherent in the MBI, and for supporting a more nuanced perspective of burnout [15,54,60,61,62].

More recently, Schaufeli et al. [63] developed yet a new measure of burnout, the Burnout Assessment Tool (BAT), to more comprehensively address various criticisms leveled at burnout measurement, including the observation of inconsistent factor structures across studies. In developing the BAT, the authors relied on a dual inductive (interviews with burnout experts) and deductive (factor analysis) approach to identify the final set of items. Notably, the BAT relies on an improved definition of burnout that excludes the professional efficacy component altogether, based on accumulating research suggesting that it is not a core component of burnout [53,64,65]. More precisely, the BAT relies on the following definition of burnout, defined as a syndrome reflecting: “a work-related state of exhaustion that occurs among employees, which is characterized by extreme tiredness, reduced ability to regulate cognitive and emotional processes, and mental distancing” [63] (p. 4). Emerging evidence already supports the psychometric properties of scores obtained on the BAT in Europe [66], Japan [67] Korea [68], and Ecuador [69]. Notably, the BAT does not present itself as a diagnostic instrument but as a screening tool to identify levels of burnout high enough to warrant further clinical assessment.

## 4. Evidence against the Distinctive Nature of Burnout

Despite the well-established nature of burnout as a work-related syndrome stemming from employees’ exposure to occupational stressors exceeding their coping abilities, uncertainty remains regarding the theoretical conceptualization of burnout, its context specificity, and its conceptual overlap with depression [13,70]. These three areas of uncertainty are intimately relevant to the core question of whether burnout should be conceptualized as a mental disorder warranting its own clinical diagnostic and are addressed in the upcoming sections.

### 4.1. Conceptual Issues

A first conceptual issue is intimately related to the popularity of Maslach et al.’s [16,20] original operationalization of burnout, which stemmed from the observation that human service employees often display unique psychological manifestations seemingly associated with their exposure to chronic levels of work-related stress. Accordingly, Maslach and her colleagues [19,20,71] sought to better capture the nature of this phenomenon through a series of in-depth interviews with human service employees. These interviews served as the basis for developing the MBI, which was then administered and factor analyzed, resulting in the three-dimensional operationalization of burnout that we know today. It would be hard to understate the merit of this initial effort, which has generated a whole new research area. However, despite this merit, it remains surprising to note that so many have simply adopted the result of this purely inductive effort as if it reflected the fundamental nature of the burnout phenomenon. If other research teams had studied this phenomenon before Maslach and her collaborators, possibly among other or more diversified populations of workers and using a more deductive process, our dominant operationalization of burnout might have been quite distinct from that underpinning the MBI [13]. Unfortunately, for many, burnout remains “what the MBI measures” rather than a clinically important psychological syndrome that can be assessed, in part, by the MBI [13]. This brings to mind a similar circularity that once plagued intelligence research.

Fortunately, despite this widespread adherence to this initial operationalization, some researchers have worked at refining, clarifying, and improving the operational definition of burnout [15,26,54,63]. However, as these efforts remain counter to the dominant approach to burnout research, consensus is still lacking. As a result, the burden of proof still lies on authors to showcase the value of these novel definitions relative to the classical MBI definition, which itself is routinely accepted as having survived the test of time. It thus remains much easier for authors to perpetuate research anchored in this classical operationalization while ignoring new developments, than to challenge this tradition. Unfortunately, this attraction to tradition perpetuates the lack of consensus regarding the optimal way to conceptualize and operationalize burnout. In a comprehensive review of the literature focused on the possible status of burnout as a mental disorder warranting a specific diagnostic category, Heinemann and Heinemann [8] noted that very few studies have attempted to develop diagnostic and differential diagnosis criteria for burnout, to identify the psychological and physical manifestations (or symptoms) of burnout, or to study biomarkers of burnout. Additionally, though many studies have sought to identify etiological causes and prevalence rates of burnout, the lack of agreed-upon diagnostic criteria means that researchers using different burnout measures could be studying different phenomenon altogether.

An arguably better alternative would be to devise a consensual clinical operationalization of burnout, anchored in a set of operational diagnostic criteria, and linked to a psychometrically validated measurement protocol. In this regard, research has generally supported many of Maslach et al.’s [16,71] early propositions (i.e., the relevance of exhaustion and depersonalization). In contrast, others have been progressively rejected (i.e., the lack of relevance of the reduced professional efficacy dimension) [64,65] or modified (i.e., the need to consider distinct types of exhaustion) [32,59]. The need to broaden our current conceptualization to better capture the whole phenomenology of burnout has also been highlighted [72]. As a result, none of the current measures seem adequate to capture the whole reality of burnout as a mental disorder linked with a distinctive clinical diagnosis. Disciplinary differences also contribute to conceptual and operational confusion. For instance, whereas psychologists seem to prefer a continuous view of burnout ranging from a complete lack of burnout to severe manifestations, medical professionals routinely prefer to define psychological difficulties as distinct categorical entities and diagnosable conditions [6]. This confusion also has practical implications, as the presence of clinically significant conditions (or diagnoses) often carries clear health-related practical and financial implications (e.g., medical leave, financial support), whereas suffering from a syndrome varying in terms of severity generally does not [6].

### 4.2. Burnout as a Work-Specific Phenomenon

Burnout was initially conceived as a work-related phenomenon [16,71], a view that still dominates the field. In fact, most of the arguments proposed to support the distinctive nature of burnout relative to other similar psychological difficulties (e.g., depression) rely on this context-specific nature of burnout [3]. In this regard, it is important to note that, although burnout is explicitly defined as an occupational phenomenon resulting from exposure to work-related stressors, it is often difficult to disentangle stress originating at work from stress originating outside of the work context (e.g., marital issues), as both typically end up interfering with both life contexts [8,12]. In addition, should this context-specificity be removed from the operational definition of burnout, then two of its key dimensions (i.e., depersonalization/cynicism at work and reduced professional efficacy) would become irrelevant, thus reducing the clinical definition of burnout to the exhaustion component alone [3,6]. Although some have argued for an exhaustion-only and context-free definition of burnout [70,73,74,75], burnout remains largely seen as a multidimensional work-specific phenomenon [3,6,8,26]. From a theoretical perspective, Schaufeli [9] further argues that both inability (exhaustion) and unwillingness (mental distancing) remain critically important facets of burnout.

Bianchi et al. [11,13] have argued against considering burnout as a distinct diagnostic category based on the rationale that its context-specificity is not inherently relevant from a nosological perspective. More precisely, they contend that the presence of context-specific causes (e.g., high job demands) does not require the development of a new diagnostic category. Instead, they suggest adapting our interventions to account for this specificity. They illustrate this argument by stating that “a job-related depression remains a depression” [11] (p. 2). They further argue that measuring burnout using an instrument specifically designed to be context-specific (such as the MBI) makes it harder to clearly assess this context specificity by making it impossible to consider that similar symptoms might, or might not, be present outside of the work context [13,76]. In other words, just because burnout was first documented among employees in their work settings does not necessarily mean it is unique to the work domain. An interesting approach to further disentangle both constructs would be to develop context-general and context-specific measures of depression and burnout to examine the extent of their overlap in a way that is disconnected from their distinctive contextual referent. In this regard, Bianchi and Schonfeld’s [77] development of a context-specific measure of depression, the Occupational Depression Inventory (ODI), represents an important first step in this direction.

### 4.3. Overlap between Burnout and Depression

The debates surrounding the context-specificity of burnout and its distinction from depression go hand in hand. Right from the start, Freudenberger [4] acknowledged that people suffering from burnout and depression look and act similarly. Though some have argued that burnout and depression differ profoundly in terms of scope (i.e., work-specific vs. general) [78], others contend that domain specificity is not sufficient for positioning burnout as its own diagnostic entity [11] for many reasons.

First, burnout and depression share a similar clinical presentation. The conceptual overlap between the manifestations of burnout and depression suggests that they may share a similar etiology [7]. This calls into question the relevance of burnout as a conceptually distinct phenomenon. In fact, some have even theorized that burnout results from unresolvable work stress [16], in the same way that learned helplessness predicts depression [79]. Indeed, both psychological constructs share core symptoms of helplessness, hopelessness, negative affect, and the absence of positive feelings [3,20,78,79,80,81]. Additional burnout symptoms have been identified with a great degree of heterogeneity, including reduced commitment and withdrawal at work, emotional reactions such as aggression and irritability, lack of interest, reduced cognitive performance, cognitive inflexibility, lack of motivation, reduced creativity and judgement, dampened emotions and social life, as well as a variety of physiological and behavioral changes [82]. Reading this list without context might lead one to assume that we describe depressive symptoms, some of which occur at work. Indeed, studies have found that individuals experiencing burnout typically also display symptoms of depression and often meet the diagnostic criteria for a depressive disorder [7,83,84,85]. Taken together, there is a substantial overlap between symptoms of depression and burnout, which is likely to be even more pronounced because of the heterogeneity and lack of consensus regarding the definition of burnout.

Second, depression and burnout are typically highly correlated with one another [85,86,87]. In particular, when assessed using the MBI, the emotional exhaustion component tends to share the highest correlations with depression [88], to the extent that it sometimes correlates more highly with depression than with the other dimensions of burnout [89,90]. However, even when the items assessing exhaustion are removed from measures of depression, burnout components have still been found to display higher correlations with depression than with one another [91]. However, the extent to which these correlations reflect conceptual overlap rather than comorbidity remains unclear and differentiating these two possibilities would require greater conceptual clarity about the validity of burnout as a distinct clinical entity.

Third, longitudinal research indicates that relations between burnout and depression are reciprocal [87,92], suggesting that suffering from burnout increases employees’ risk of depression, while depression also increases their risk of burnout. Two conjectures surround these reciprocal associations [11,93]. One suggests that burnout represents an early, context-specific phase in the development of depression, progressively interfering with all spheres of life. The other suggests that depression has a maladaptive impact on employees’ work experiences, thus making them more likely to experience burnout. Clearly, more research is needed to better understand the mechanisms, whereby burnout and depression progressively increase the risk of experiencing the other. However, additional studies will first require clarification of whether these constructs are truly distinct.

## 5. Evidence Supporting the Distinctive Nature of Burnout

Despite the aforementioned criticisms, many still consider burnout to be meaningfully distinct from depression. For instance, Epstein and Privitera [94] argued that, although evidence supports the presence of substantial overlap between both constructs, workers do not always simultaneously experience burnout and depression. They further highlight that, relative to depression, burnout cannot be considered a purely individual syndrome because it is intimately related to a “breakdown in the relationship between people and their work” [94] (p. 1). From a practical perspective, they note that conceptualizing burnout as depression would stigmatize the struggles of many employees from occupational groups particularly affected by burnout (e.g., physicians) as an individual issue, rather than properly contextualizing their distress within a problematic systemic or institutional setting [94]. To address these concerns, Bianchi et al. [32] noted that the reliance on a measure of depression specific to work would make it possible to retain these contextual considerations. However, this recommendation relies on the assumption that burnout and depression are empirically indistinguishable phenomena.

In a meta-analysis of research published between 2007 and 2018 to examine the overlap between burnout, depression, and anxiety, Koutsimani et al. [89] noted that, although there was indeed evidence of overlap between burnout, depression, and anxiety, the bulk of the results still supported the distinctive nature of these three psychological states. Interestingly, they also found that studies using the MBI tended to demonstrate cleaner distinctions between the constructs relative to those relying on other measures of burnout. The distinction between burnout and depression has also been supported by various factor analytic studies supporting the idea that both constructs retain a meaningful amount of specificity once their common core is considered [63,64,87]. There is also qualitative evidence for the distinctive nature of burnout and depression. For instance, Tavella and Parker [95] recruited 1019 workers who self-reported having experienced both depression and burnout. These workers completed an open-ended questionnaire recording the symptoms they attributed to burnout, the main cause of their symptoms, and how they differentiated between burnout and depression symptoms. Their qualitative analyses revealed that several themes contributed to differentiate burnout from depression. Thus, whereas participants were able to ascribe a clear external cause to their symptoms of burnout, they had a more challenging time identifying the causes of their depression, which seemed to be more intrinsically rooted. Participants also reported that burnout was associated with higher functioning, greater self-esteem, less suicidal ideation, and more hope than depression. Additionally, participants reported that burnout felt more like an anxious and activated state than depression, which was characterized by feelings of heaviness and slowness. The presence of anhedonia in depression, but not in burnout, was another major distinction. Finally, approximately one-fifth of the participants could not distinguish depression from burnout, with some noting that their experience of burnout led to depression.

Lastly, in a recent longitudinal investigation of the reciprocal associations between burnout and depression, Tóth-Király et al. [92] found evidence supporting the discriminant validity of both constructs. First, they demonstrated that while burnout was an inherently multidimensional construct, depression was better conceptualized as a unidimensional phenomenon. Second, although both constructs were found to be reciprocally related both within and over time, their associations remained moderate in magnitude, consistent with the presence of variability uniquely associated with both constructs. Lastly, they found evidence that both constructs shared a well-differentiated pattern of association with a series of theoretically relevant covariates.

## 6. Burnout as a Diagnostic Category

To summarize the previous discussion, current theoretical considerations and empirical results can be leveraged to support both the notion that burnout is distinct from depression and that these two psychological phenomena share substantial conceptual overlap. Based on this conflicting evidence, some researchers argue that burnout is simply work-specific depression and should be studied as such [13,77,88,96], whereas other argue that both represent theoretically and empirically distinct psychological constructs [89,92,97,98]. Nonetheless, while the burnout–depression debate is an important and pervasive one, it is not the only consideration from a diagnostic perspective. To determine whether burnout should or could conceivably be characterized as a mental disorder, burnout must also be evaluated within the context of current diagnostic classification models. This examination might help us to devise the next steps in a research program that could also prove helpful in clarifying the burnout–depression debate.

In and of itself, the debate surrounding the classification of burnout as a diagnosable condition is not new. Though no universally agreed-upon definition of burnout exists and though burnout has not yet been formally incorporated in any existing diagnostic classification systems, researchers and clinicians have been classifying and “diagnosing” burnout as a syndrome for years [82]. This may be related, at least in part, to the fact that in the early days of burnout research, Maslach et al. [99] included cut-off scores in the MBI test manual to help identify clinically significant levels of burnout. Thus, although the MBI was explicitly designed as a continuous measure of burnout severity, these cut-off scores sparked a surge of studies in which employees were classified based on the severity of their symptoms. Additionally, because accumulated research evidence supports the clinical validity and utility of the MBI, some researchers have concluded that it was therefore suitable as a diagnostic tool [49,100]. However, measures of burnout rely predominantly, if not exclusively, on self-report. This is, and has always been, a considerable limitation of psychological or psychiatric diagnoses, which cannot be established in the absence of additional signs of health and behavior typically collected via clinical interviews [21]. One last consideration comes from the more practical considerations of health professionals, whose clinical services are typically more easily reimbursed by governmental agencies or insurance companies when there are linked to diagnosable medical conditions [3].

Nowadays, several European countries acknowledge burnout as a legitimate mental disorder. Lastovkova et al. [14] and Guseva Canu et al. [101] contrasted burnout classifications and found that it is acknowledged as an occupational disease in 14 European countries but is only officially listed as an occupational disease in Latvia. In five European countries, it is common practice to grant workers suffering from burnout with financial compensation. In Iceland, the Netherlands, and Sweden, if one can prove causality related to work conditions, any illness or injury can be classified as an occupational disease, including burnout [14,101]. The Netherlands is currently the only country using the MBI as a clinically validated diagnostic tool, issued by the Royal Dutch Medical Association as a strategy to manage stress-related disorders [102]. Diagnostic criteria vary widely by country, with most criteria derived from regional or national committees [14,101]. Thus, although burnout is a common diagnosis in Europe, there is currently no consensus surrounding financial compensation, diagnostic criteria or protocols, and the role of work-related and individual determinants in the etiology of burnout [101].

### 6.1. Primary Classification Systems

Turning our attention to primary diagnostic systems, the Diagnostic and Statistical Manual for Mental Disorders (DSM-5; APA, 2013) and the International Classification of Diseases [103] are currently the most frequently used diagnostic classification systems internationally. They define mental disorders as the presence of significant impairments in functioning across contexts in the areas of cognition, emotion regulation, and/or behavior [80,103]. Both systems seek to provide a common vocabulary to improve diagnostic accuracy, public understanding, and treatment accessibility [80,103,104]. To be diagnosed with any mental disorder, one must exhibit a particular set of symptoms. Such criteria are determined by committees comprised of researchers and mental health professionals, who iteratively make decisions based on evidence derived from empirical studies and clinical trials [80,102,103].

Burnout is not currently characterized as a mental disorder or medical condition in the DSM-5 [80]. However, the World Health Organization recognizes burnout as an important occupational phenomenon under the category of “factors influencing health status or contact with health services” in the ICD-11 [103]. The ICD-11 defines burnout as a syndrome resulting from chronic work stress, in a way that matches Maslach’s original conceptualization [20]. The ICD-11 also states that burnout should not be identified in the presence of adjustment, stress-related, anxiety, or mood disorders.

To be included in these primary diagnostic systems, in alignment with the medical model which underpins them, one must be able to demonstrate that new disorders can be linked to consistent etiological risk factors, pathological processes, symptom patterns, concurrent validators, and comorbidities, while accounting for a variety of cultural, social, psychological, and developmental considerations [80,104,105]. Clark et al. [104] identified four key issues related to the classification of mental disorders. First, the drive to find clear causes for mental disorders, in the same manner as physical health conditions, is complicated by the fact that psychopathologies are typically influenced by a complex pattern of interaction among a variety of biological, behavioral, psychosocial, and cultural factors, making their etiology more complex to uncover than that of purely physiological medial conditions. Second, the categorical nature of these systems oversimplifies the fact that mental disorders involve complex combinations of dimensional problems (i.e., varying in terms of severity rather than in terms of presence or absence) that are often transdiagnostic (i.e., play a role in many mental disorders). Third, because mental disorders are typically multidimensional (i.e., entailing a combination of behavioral, cognitive, emotional, and physical manifestations), setting diagnostic thresholds, and making decisions regarding the clinical significance of symptoms tends to be very difficult in practice. Finally, categorical classification systems face the problem of artifactual versus actual comorbidity. In other words, patients often display symptoms associated with various disorders, thus decreasing diagnostic accuracy and clinical utility [104]. Incorporating new disorders into these primary diagnostic systems thus entails years of research focusing on these complex considerations [105].

### 6.2. Evaluation and Future Directions

Based on the aforementioned considerations, characterizing burnout as a mental disorder within these existing classification systems does not currently seem appropriate. First, the rigorous empirical evaluation of burnout required for diagnostic classification is muddied by various longstanding conceptual and measurement issues/inconsistencies [13]. Without a clear definition anchored in a consensual set of symptoms, it remains impossible to establish reliable prevalence rates or properly compare etiological and outcome-related findings across studies [6,12]. Determining clear etiology for mental disorders is complicated in general [104,105] and even more so when phenomena lack a clear definition to begin with [12]. Thus, before considering burnout for inclusion in any existing diagnostic classification systems, conceptual inconsistencies must be clarified, and consensus must be reached regarding how to define and measure burnout. While efforts to arrive at a consensus have historically triggered more research using the MBI [13], thus reifying the same conceptual definition in a way that some have described as circular, the development and emerging evidence of validity associated with the BAT offers some promise in this area.

Second, the medicalization of burnout would entail a shift away from a continuous (i.e., varying in terms of severity) toward a dichotomous (i.e., burned out or not burned out) or categorical (i.e., cut-off scores for low, medium, and high levels of burnout) conceptualization of burnout [6]. On the one hand, insisting that burnout is best defined as a multidimensional continuous phenomenon is inherently incompatible with our current categorical diagnostic systems. On the other hand, there is currently no standard definition of what being “burned out” truly means from a dichotomous or categorical perspective. Though it is often reduced to exhaustion alone [73,74,75,106], Schaufeli [9] contends that the core of burnout entails both the inability (exhaustion) and unwillingness (mental distance) to work. Thus, for burnout to be integrated into an existing diagnostic system as a separate diagnosis, we first need to reach a consensus on this matter.

Third, we also currently lack consensus regarding which specific symptoms should be used to define burnout and on how these symptoms differ from those of other well-understood mental disorders. As noted previously, there is a great degree of heterogeneity in the burnout symptoms identified in the current research literature [82], and many of these symptoms share a substantial degree of overlap with those of depression [3,20,78,79,80,81]. As such, an important step would be to develop clear and widely agreed-upon set of diagnostic criteria (i.e., symptoms), diagnostic thresholds, and differential diagnostic criteria. Additionally, we will need to determine whether the high comorbidity typically reported between depression and burnout is artifactual (i.e., burnout is simply work-related depression) or whether they represent meaningfully distinct constructs that constitute different diagnostic categories. Longitudinal investigations, such as that conducted by Tóth-Király et al. [92], would be helpful in this regard, making it possible to systematically assess the nature of the overlap between both phenomena, as well as the similarities and differences in their etiology and implications. Likewise, person-centered analyses [107] would also help to systematically capture subpopulations of individuals presenting overlapping and non-overlapping conditions.

Fourth, research evaluating burnout’s neural, biological, genetic, and cognitive correlates remains underdeveloped [8], which represents an important obstacle to positioning burnout as a distinct diagnostic category within a medically inspired classification system. In this regard, Bayes et al.’s [108] systematic literature review revealed that burnout was potentially associated with autonomic nervous system activation, changes in cortisol levels, immune functions, and endocrine functions. However, the lack of consensus related to burnout definition and measurement, variability in results across distinct populations, and the predominantly cross-sectional nature of the studies made it difficult to draw any firm conclusions [108]. Homogenous clinical samples and longitudinal studies would be needed to determine biomarkers able to differentiate burnout from other well-established mental disorders [109].

Lastly, the relevance of positioning burnout as a mental disorder should ideally be investigated from a transdisciplinary angle that includes both clinical and social psychologists, as well as non-academic stakeholders. Currently, it appears that research is being conducted in isolation, raising questions about possible confirmation biases. Similarly, although burnout is not currently categorized as a mental disorder and has no clear diagnostic criteria, individuals in some countries are still being classified as suffering from burnout and treated for their condition. Research has shown that it is difficult to objectively compare burnout interventions as they vary considerably but that individual-focused interventions are not necessarily sufficient [110,111]. In this regard, combination therapy may be an effective avenue to addressing both the individual (e.g., coping-related) and environmental (e.g., job demands and social support) origins of ‘burnout’.

## 7. Conclusions

After decades of research, burnout can be considered to reflect a widely accepted syndrome stemming from employees’ persistent exposure to work-related stressors while lacking the necessary resources to efficiently cope with these stressors [6,15,16,71]. This syndrome is typically defined as a state of psychological and physiological exhaustion and disengagement from work [9,15,63], although no consensus currently exists regarding its other defining characteristics [64,65,95]. Despite the joint recognition of burnout as an important psychological phenomenon by researchers, professionals, and the public alike, there is also an ongoing debate regarding whether burnout should be characterized as a distinct mental disorder [8]. As such, although burnout has been intensively researched for decades, the bulk of burnout research documents its relevance as a work-related psychological syndrome with severe consequences for employees and organizations, rather than seeking to isolate its distinctive biopsychosocial etiology, categorical nature, discriminant validity in relation to other mental disorders, and comorbid versus overlapping nature with these other disorders.

More precisely, the lack of conceptual clarity and consensus how to define and measure burnout means that we do not have reliable prevalence rates, etiology, diagnostic features/criteria, differential diagnostic criteria, or biomarkers [6,8,12,13,104]. Additionally, ongoing debates surrounding the distinction between burnout and depression and whether the work-specific nature of burnout is sufficient to warrant its own diagnostic category [7,11,13,76] remain unresolved. In sum, there is presently no definitive evidence supporting or failing to support the relevance of burnout as a diagnostic category, leaving the field with more questions than answers. Given the current state of research in this area, it would thus seem inappropriate, or at least hasty, to incorporate burnout as a distinct mental disorder into any existing diagnostic classification systems.

In contrast, despite some criticisms, the current state of evidence seems to support the relevance of considering burnout as a psychological syndrome, varying in severity. Although debates and confusion remain, particularly when considering whether and how burnout differs from depression, it would seem premature to discard burnout altogether and to replace it with an occupational depression syndrome. Indeed, over time, research evidence has established the heuristic value of burnout as a useful component in research and practice seeking to understand the process whereby employees cope, or fail to cope, with work-related stress. Moreover, meta-regression analyses conducted on the results from 69 studies has recently shown that using a single point estimate to conceptualize the empirical relationship between the two may mask a more complex picture [108]. Additional research is required to better document its scientific relevance and its distinctive nature and etiology. From our standpoint, this would be best accomplished by capitalizing on the already established heuristic value of burnout as a psychological syndrome to start more extensive consensus-building efforts. Burnout is too deeply entrenched in our cultural experience and vernacular, playing a critical role in conversations about public wellbeing [8], to be discarded without further research seeking to better establish its distinctive nature.

Only after clarifying these core remaining issues would it become relevant to open up, once again, the question of whether burnout should be considered as a mental disorder warranting its own diagnostic category. Above all else, conceptual and measurement problems would need to be addressed in a cross-national and collaborative manner, seeking to clarify its definition, core symptoms, and biopsychosocial etiology, while avoiding the trap of reifying old definitions as if they did reflect some observable reality [8]. Classical definitions and seminal measures such as the MBI should be acknowledged for the important role they played in stimulating decades of incredibly rich research efforts, rather than as a definitive way to study a phenomenon that has not yet been clearly operationalized. Doing so will provide a more solid theoretical basis for longitudinal and prospective empirical studies to identify causes and outcomes, biomarkers, cultural variations, and associations with other similar constructs.

## Data Availability

No data was used in the preparation of this review paper.
